# Green Space and Stress: Evidence from Cortisol Measures in Deprived Urban Communities

**DOI:** 10.3390/ijerph10094086

**Published:** 2013-09-02

**Authors:** Jenny J. Roe, Catharine Ward Thompson, Peter A. Aspinall, Mark J. Brewer, Elizabeth I. Duff, David Miller, Richard Mitchell, Angela Clow

**Affiliations:** 1 School of the Built Environment, Heriot-Watt University, Edinburgh EH14 4AS, UK; E-Mail: p.a.aspinall@hw.ac.uk; 2 OPENspace Research Centre, University of Edinburgh, Edinburgh EH3 9DF, UK; E-Mail: c.ward-thompson@ed.ac.uk; 3 Biomathematics and Statistics Scotland, Aberdeen AB15 8QH, UK; E-Mails: m.brewer@bioss.ac.uk (M.J.B.); e.duff@bioss.ac.uk (E.I.D.); 4 James Hutton Institute, Aberdeen AB15 8QH, UK; E-Mail: david.miller@hutton.ac.uk; 5 Centre for Research on Environment, Society and Health, Institute of Health and Wellbeing, University of Glasgow, Glasgow G12 0XH, UK; E-Mail: richard.mitchell@glasgow.ac.uk; 6 Department of Psychology, University of Westminster, London W1B 2UW, UK; E-Mail: clowa@westminster.ac.uk

**Keywords:** green space, stress, diurnal, saliva, cortisol, neighbourhood, urban, deprivation, gender, mental health

## Abstract

Contact with green space in the environment has been associated with mental health benefits, but the mechanism underpinning this association is not clear. This study extends an earlier exploratory study showing that more green space in deprived urban neighbourhoods in Scotland is linked to lower levels of perceived stress and improved physiological stress as measured by diurnal patterns of cortisol secretion. Salivary cortisol concentrations were measured at 3, 6 and 9 h post awakening over two consecutive weekdays, together with measures of perceived stress. Participants (n = 106) were men and women not in work aged between 35–55 years, resident in socially disadvantaged districts from the same Scottish, UK, urban context as the earlier study. Results from linear regression analyses showed a significant and negative relationship between higher green space levels and stress levels, indicating living in areas with a higher percentage of green space is associated with lower stress, confirming the earlier study findings. This study further extends the findings by showing significant gender differences in stress patterns by levels of green space, with women in lower green space areas showing higher levels of stress. A significant interaction effect between gender and percentage green space on mean cortisol concentrations showed a positive effect of higher green space in relation to cortisol measures in women, but not in men. Higher levels of neighbourhood green space were associated with healthier mean cortisol levels in women whilst also attenuating higher cortisol levels in men. We conclude that higher levels of green space in residential neighbourhoods, for this deprived urban population of middle-aged men and women not in work, are linked with lower perceived stress and a steeper (healthier) diurnal cortisol decline. However, overall patterns and levels of cortisol secretion in men and women were differentially related to neighbourhood green space and warrant further investigation.

## 1. Introduction

Contact with green space has been associated with benefits to mental health, particularly levels of stress [1]. These effects are thought to operate through one or more of three mechanisms: increased physical activity (see [2] for a systematic review) which, in turn, improves mood [3]; increased social contact and sense of “belonging” within a community [4]; and psychological restoration related to attention processes and attenuation of stress and fatigue [5,6]. Two theoretical models of psychological restoration have been proposed. The first is related to the Kaplans’ Attention Restoration Theory [6] which posits that natural settings, inherently rich in stimuli, invoke involuntary attention which supports restoration from mental fatigue. The second is Ulrich’s psychoevolutionary model [7] which posits that stress reduction arises from an immediate, affective response to the visual stimulus array of a natural setting, impacting the brain and neuroendocrine system. However, evidence of an impact from contact with natural environments on the neuroendocrine system is currently limited to a small number of studies.

Contact with nature has been shown to have positive effects on blood pressure [5,7], heart rate [7], skin conductance and muscle tension [7]. However, most studies which have used salivary cortisol as a measure of stress in relation to contact with nature have not measured diurnal patterns, focussing instead on measuring levels immediately before and after exposure to different environmental settings. For example, Park and colleagues [8] have shown visiting a Japanese forest can reduce cortisol concentrations, compared to city environments. Lee *et al*. [9] reported that visiting a forest had greater benefits for stress levels than visiting an urban environment, using cortisol and pulse rates as biomarkers of stress. Similarly, van den Berg and Custer [10] found that a period of allotment gardening led to greater levels of stress reduction than a restful indoor task (*i.e.*, reading), using salivary cortisol as a measure. Although these studies found that contact with natural environments reduced stress, their external validity is limited because they either had very small numbers of participants or limited categories of participant (e.g., university students, allotment gardeners) or used very controlled, specific settings (possibly both). Very little research has examined the stress neuroendocrine system in relation to the long-term effects of familiar, everyday environments, set within the context of people’s normal patterns of activity and experience. One exception is the study by Do *et al*. [11] which explored the relationship between a series of neighbourhood characteristics (deprivation, violence, disorder and social cohesion) and changes in diurnal cortisol rhythms. In that study, higher perceived levels of neighbourhood violence were significantly associated with lower cortisol values on awakening and with a less steep decline in cortisol concentrations from the morning to the evening.

Some evidence points to demographic and socio-economic differences in the relationship between green space and health. An inverse association between green space and all-cause mortality rate appears stronger for more deprived populations than for more advantaged populations in England and Wales [12]. Greater beneficial effects of local green space have been found in people spending more time in their local neighbourhood (e.g., young people, older people, people not in work), perhaps due to greater exposure to this green space [13]. Gender differences in the relationship between environmental factors and health outcomes are beginning to emerge but remain under-explored. For example, a population-level study in the UK has shown gender differences in the relationship of urban green space to cause-specific mortality rates [14]. The authors suggested this difference was due to gender differences in perceptions and usage of urban green space. Fear for safety is a common barrier experienced by women in accessing urban green space [15] and a dimension that reduces the likelihood of women walking in their local neighbourhoods, as compared to men [16]. A wider general literature that has explored gender differences in the impact of general neighbourhood context on health has generally found stronger effects on women than men [14].

Gender differences in the relationship between stress and the diurnal pattern of cortisol secretion have also been reported. In middle-aged men, stress has been associated with higher cortisol concentrations and a reduction in the diurnal cortisol decline (henceforth referred to as cortisol slope): a “high and flat” pattern. In contrast, stressed middle-aged women presented with low cortisol concentration and a lower and flatter cortisol slope [17]. Consistent with this finding, in older women depression has been associated with hypocortisolemia whilst, in older men, depression was associated with hypercortisolemia [18]. Lower cortisol levels have also been linked with negative life events, associated with significantly lower cortisol levels in females [19]. There is increasing evidence for a life-course influence on the cortisol cycle. For example, early life adversity has been shown to be associated with lower cortisol profiles [20,21]. In summary, there appear to be gender differences in the impact of neighbourhood characteristics on health as well as gender differences in the neuroendocrine response to negative life events and stress.

This study builds on an earlier, exploratory study [22] of a socially deprived urban population that found significant relationships between measures of neighbourhood green space, perceived stress and the diurnal pattern of cortisol secretion. Higher levels of neighbourhood green space were linked with lower levels of perceived stress and a steeper diurnal decline in cortisol secretion. In that study, as in this one, neighbourhood was defined using Census Area Statistics (CAS) Ward. This is a small areal unit used in the administration of the UK decennial census. It has previously been used as a definition of neighbourhood in other research on green space and health [14,23]. Based upon this earlier study, we generated two hypotheses for the current study:

1. *Hypothesis 1*: greater availability of neighbourhood green space in deprived urban communities is linked to lower perceived stress;
2. *Hypothesis 2*: greater availability of neighbourhood green space in deprived urban communities is linked to lower levels of physiological stress, as measured by the diurnal patterns of cortisol secretion.

Based upon the accumulating evidence cited above indicating gender differences in stress responses we expanded the present study to include a third hypothesis:

3. *Hypothesis 3*: patterns of cortisol secretion in this socially deprived urban population will be differentially expressed in men and women. We did not predict what pattern these would follow by levels of neighbourhood green space.

## 2. Experimental Section

### 2.1. Study Design

The study repeats the earlier study [22] in outcome measures, demographics, geographic location and cross-sectional design. As before, it included a survey of participant demographics, perceived stress, well-being and self-reported exercise levels. Repeated salivary cortisol sampling took place over two consecutive weekdays. The study employed secondary data analysis of participants’ local environments in order to determine percentage of green space in each participant’s neighbourhood. The study was set in the city of Dundee, United Kingdom, selected because it contains a number of highly deprived urban neighbourhoods with varying levels of green space. The study was carried out in accordance with the British Psychological Society “Ethical Principles for Conducting the Research with Human Participation” and with full ethical approval granted by the lead researchers’ institutional ethics board.

### 2.2. Participants

To reduce age-related effects on patterns of cortisol secretion our participants were men and women aged 33–55 years of age. They were not in work for any reason (e.g., job-seeking unemployed, on invalidity benefit, carers) and were living in socio-economically deprived areas of Dundee as measured by the Carstairs Index of deprivation [24]. The Carstairs Index is a widely used and well-validated indicator of area-level socio-economic deprivation based on prevalence of household overcrowding, unemployment among men, low social class, and not having a car; the higher the Carstairs score, the greater the deprivation. Participants were recruited from areas with a Carstairs score of 5–7 (the average Carstairs score for Dundee is 3.93).

### 2.3. Recruitment

Recruitment was through a two-stage process. Firstly, specified postcode areas in the city of Dundee were selected on the basis of Carstairs indices of 5–7. Within these areas a campaign of door-to-door recruitment was undertaken over a period of five weeks, from May to June 2010. Interested participants that fulfilled the inclusion criteria were provided with information sheets and, following an indication that they were willing to take part, their details were recorded by the recruitment company and followed-up by the by the research team. Potential participants were invited to a group briefing session in the city-centre. People were excluded if they were taking oral steroids, but more stringent exclusion criteria (e.g., use of anti-depressants, smoking) although recorded, were waived owing to the high likelihood of finding such behaviour patterns in the target sample population. People who had lived for less than 12 months in their neighbourhood were also excluded.

At the group briefing session, following informed consent, participants completed a short, paper-based questionnaire and were instructed on the protocol for taking cortisol samples. Of the 158 potential participates that attended the briefing session 81 participants successfully completed the study. As statistical analyses established no effects of season or demographic differences between this sample and the exploratory study sample (gathered in January 2010), the databases were merged to achieve greater statistical power with a total sample size of 106 participants, 50% male and female.

### 2.4. Measures

The primary outcome measures were perceived stress (the Perceived Stress Scale (PSS) [25], the cortisol slope and average cortisol levels, derived from multiple saliva sampling across two consecutive days. The PSS comprises 10 items measured on a 5-point response from “never” to “very often”. The final score assesses perceived stress over the preceding month and can range from 0 (minimum level of stress) to 40 (maximum level of stress). The detailed procedure for the cortisol sampling is described below.

Other measures included positive well-being and self-reported levels of physical activity, captured as potential explanatory variables. Well-being was measured using a shortened version of the Warwick and Edinburgh Mental Well-being Scale (SWEMWBS) [26]. SWEMWBS asks participants how they had felt over the previous four weeks in relation to seven items used to measure aspects of mental well-being (e.g., feeling relaxed, feeling useful), with responses rated on a 5-point scale from “*none of the time*” to “*all of the time*”. Final scores can range from 7 (low well-being) to 35 (high well-being). Physical activity was measured using one item asking for the number of days on which physical activity (of sufficient exertion to raise breathing rate) reached or exceeded 30 min, recalled over the past four weeks. This item is recommended by the British Heart Foundation National Centre [27].

Socio-economic deprivation was based on the Carstairs Index for population data in 2001 [24], obtained via each participant’s postcode. Participants’ age and gender were also recorded.

Exposure to green space was measured by the percentage of the area in the Census Area Statistics (CAS) Ward of the participant’s residence which was identified as green space. The green space measure includes parks, woodlands, scrub and other natural environments, but not private gardens (although participants indicated yes/no as to whether they had access to a garden). Dundee contains 31 CAS Wards with a mean population of 4942 at the 2001 census, and a mean proportion of green space of 33.89%. These data were created by the Centre for Research on Environment Society and Health (CRESH) [14,23] and are freely available at the CRESH website [28].

### 2.5. Cortisol Sampling Procedure

Participants were instructed to take three samples of saliva per day on two consecutive weekdays (Monday and Tuesday) using salivette saliva sampling devices (Starstedt, Leicester, UK). Requested sampling times were 3, 6, and 9 h after awakening. They were instructed not to smoke, eat or drink anything but water 30 min before taking each sample and to keep a log of sample times. Wake up time was defined as the moment a participant was first conscious of being awake. To maximize adherence, participants were sent individualised SMS text prompts, based upon self-report predicted awakening times, 3 times on each day to remind them to take their samples. At the end of the day, participants were asked to freeze their samples (or place in a fridge) in a sealed bag provided. They were then collected and shipped to the laboratory for assay analysis within five days of collection. Cortisol assays were carried out by Salimetrics Europe using Enzyme Linked Immuno-Sorbent Assay. The lower limit of sensitivity is 0.01638 nmol/L. The standard range in assay is 0.513–8.468 nmol/L; correlation of assay with serum: r (47) = 0.91, *p* < 0.0001; intra and inter-assay variations were both below 10%. Average daily cortisol concentrations were determined by calculating the average cortisol concentration across all sampling points on both sampling days. The cortisol slope was derived from the change in cortisol concentration from 3–9 h post awakening, averaged across both sampling days. The use of start and end data points to determine the linear slope follows methods by Thorn *et al*., [29] and Power *et al*., [30].

### 2.6. Data Cleaning

When computing cortisol regression analyses, a total of 13 participants were initially removed for data quality issues (*i.e.*, a cortisol sample outside the required time threshold of plus or minus 45 min from target sampling time, and/or missing data). Any single measurement greater than two standard deviations from the mean was retained with that measure winsorised. This resulted in a sample size of 93 for mean cortisol; for cortisol slope (requiring means extant for: sample times 3, 6 and 9 h), five further participants were removed owing to doubts about the timing of the sampling (less likely to affect the mean cortisol), giving a sample size of 88. For the perceived stress regression, three participants were removed for data quality issues, resulting in sample size of 103. When computing descriptive statistics, a total of two participants were removed owing to missing data, resulting in a sample size of 104. In summary, data were cleaned or participants removed in preparation for four different analyses: cortisol slope, cortisol mean; perceived stress; and descriptive statistics.

### 2.7. Statistical Analysis

Bivariate relationships between variables were explored using Pearson correlations. Gender differences in socio-demographic characteristics and outcome variables were assessed via Mann-Whitney tests, as the data were not normally distributed. Multiple linear regressions examined main effects and interaction effects in relationships with the primary outcome variables of perceived stress, cortisol slope and average cortisol concentrations, and the predictors of age, gender, deprivation level, physical activity level and the amount of neighbourhood green space; this selection of covariates is justified by the findings of our earlier study. Since model diagnostics found the average cortisol concentration to violate the assumption of normality, a log-transformed variable was used to remove this. Variance inflation factors confirmed there were no collinearity issues in all final models presented.

In initial regressions, perceived income coping was found to be a very high correlate of perceived stress (*i.e.*, the two share a very strong relationship with each other, in suggesting that coping with low income levels is likely to be a main cause of stress in people living with unemployment). As a consequence, in this particular data set, income coping can be regarded as a surrogate for stress, and was therefore removed from the analysis. Physical activity was not significantly associated with outcome measures in exploratory analyses, and was removed from the models for this reason. It is possible that the number of missing values with respect to physical activity (giving a smaller resultant sample of 88) may account for this. We found a similar pattern with the life events scale, *i.e.*, it was not a significant predictor of stress in exploratory regression analyses, but 25% of participants did not answer this question. We note that removing variables from models in this manner can lead to “omitted variable bias”, so in each case we checked that the coefficients of remaining variables were not altered greatly as a result.

Relationships between outcome measures and green space were examined carefully; while a linear relationship seemed appropriate for two of the measures (perceived stress and mean cortisol), there did seem to be evidence of a step-change with respect to cortisol slope, suggesting that a binary indicator might be more suitable than a straight line. We required an objective, scientific method for determining where on the continuous scale to make the split into two (or more); for this purpose we used SPSS AnswerTree to establish an optimal split of 43% green space using a CHAID (CHI squared Automatic Interaction Detector) analysis. Note further: the algorithm used had the potential to make single or multiple splits, but in the event, only one was found to be significant (at 43%). The term “low green space” henceforth refers to areas with less than (or equal to) 43% green space; the term “high green space” to areas with more than 43% green space. While associations between stress levels and access to private or semi-private gardens are not a focus of this paper, we felt it important to ensure any effects of a household having access to a garden were accounted for prior to assessing any effects of access to publicly accessible green space. Reported access to a garden (yes/no) was therefore included as a co-variate in the regression analyses.

## 3. Results and Discussion

### 3.1. Results

A repeated measures ANOVA factoring day (sampling days 1 and 2), and sample (cortisol concentrations at 3, 6, 9 h post-awakening) revealed no significant main effect for day. As expected, there was a highly significant main effect of sampling time (F = 26.62, df = (1,54), *p* < 0.001), indicating that cortisol means varied across the day. Both results suggested participant adherence to the required sampling protocol and legitimised the strategy of averaging both cortisol measures (average concentration and cortisol slope) across the two sampling days to give the most reliable measures.

The demographic profile of participants is provided in Table 1. Women reported higher perceived stress, lower well-being and lower levels of physical activity and their average cortisol concentrations were significantly lower than men’s.

**Table 1:** Characteristics of the study population (n=104).

							Total Mean (SD)	Male Mean (SD)	Female Mean (SD)
*Percentage sample*								50%	50%
*Mean age* (years)							44.75 (6.91)	44.21 (6.65)	45.28 (7.20)
*Level of deprivation (* mean Carstairs score)							6.64 (2.21)	6.28 (2.36)	7.0 (2.02)
*Work status %*									
	Working part-time (less than 30 h/week)						3	2%	4%
	Job-seeking						67%	79%	57%
		In education					4%	4%	4%
			Not in work owing to sickness, disability				11%	11%	11%
				Not in work owing to caring for family			13%	4%	22%
					Unknown		1%	0%	2%
*Percentage of green space in residential environment*									
						Low green space < 43%	73%	77%	70%
						High green space ≥ 43%	27%	23%	30%
Perceived Stress (PSS)							14.28 (5.8)	18.66 (5.9) *	21.87 (5.40) *
Perceived well-being (SWEMWBS)							22.35 (4.94)	23.55 (4.92) *	21.10 (4.67) *
Reported physical activity (days/month)							8.20 (8.71)	9.70 (8.9) *	6.67 (8.29) *
Cortisol mean concentration (over 2 days)							6.00 (2.85)	6.54 (2.54)	5.58 (3.08)
Cortisol slope (mean slope over 2 days)							4.00 (5.00)	4.09 (4.74) *	3.91 (5.27) *

* statistically significant gender difference between male and females *p* < 0.05 (Mann-Whitney U Test).

Bivariate correlations revealed that, within the total population, higher mean cortisol concentrations were positively associated with a higher cortisol slope (Table 2). This atypical relationship indicates that, in this socially disadvantaged sample, low cortisol levels were associated with flat diurnal cortisol profiles, *i.e.*, low cortisol levels were associated with less healthy cortisol profiles. The cortisol slope was negatively associated with perceived stress and positively associated with physical activity and more green space (measured on the binary variable). Higher percentage neighbourhood green space was associated with lower perceived stress and higher mean cortisol secretion.

Gender differences in stress levels were found when taking account different levels of green space in the residential environment (Table 3). In both men and women, perceived stress was higher in low green space areas, but women’s perceived stress was significantly higher in low green space areas than men’s (*p* < 0.05). Also, women had significantly lower mean cortisol concentrations in lower green space areas compared to men (*p* < 0.05), indicative of greater hypocortisolemia (low flat cycle). It is notable that the differences in stress levels between men and women were only significant in low green space areas, with evidence of greater comparability in stress levels in high green space areas.

**Table 2:** Relationships between cortisol patterns, health measures and percentage green space.

	*Correlations between variables*						
	1 Cortisol mean	2 Cortisol Slope	3 Stress (PSS)	4 Well-being	5 PA	6 % GS	7 Bin GS
1. Cortisol Mean	1						
2. Cortisol Slope	0.458 ***	1					
3. Stress (PSS)	−0.112	−0.173 *	1				
4. Well-being (SWEMWBS)	0.099	0.109	−0.616 ***	1			
5. PA (Physical Activity)	0.113	0.207 *	−0.069	0.003	1		
6. Percentage Green Space (% GS)	0.311 ***	0.121	−0.286 **	0.080	−0.014	1	
7. Binary green space (BinGS)	0.136	0.174 *	−0.144	0.107	−0.033	0.812 ***	1

* *p* < 0.10; ** *p* < 0.05; *** *p* < 0.01. The reported correlation coefficients are Pearson’s (relatively similar Spearman rank correlations were found). Log transformed data was used for cortisol mean analysis.

**Table 3:** Gender differences in stress levels by level of green space.

Variable	Male mean (SD)		Female mean (SD)	
	*Low Green Space*	*High Green Space*	*Low Green Space*	*High Green Space*
Perceived Stress (PSS)	13.24 * (5.80)	10.92 (5.83)	16.49 * (5.94)	14.38 (3.38)
Cortisol mean concentration (nmol/L) (over 2 days)	7.62 * (10.22)	4.61 (1.14)	4.24 * (2.03)	6.43 (3.50)
Cortisol slope (nmol/L) (mean slope over 2 days)	3.87 (4.89)	4.98 (4.16)	3.25 (4.49)	5.67 (6.85)

* statistically significant difference between males and females *p* < 0.05 (Mann-Whitney U Test).

Regression analyses were performed to predict perceived stress, the cortisol slope and average cortisol secretion over the day (see Table 4).

**Table 4:** Linear regression model analyses predicting perceived stress, cortisol slope and cortisol mean over 2 days.

Main Effects	Model 1 Perceived stress (n = 103)			Model 2 Cortisol Slope (n = 88)			Model 3 Cortisol mean (n = 93)		
Variable	β	t	95% CI	β	t	95% CI	β	t	95% CI
Gender (female)	5.47 ***	3.11	1.98, 8.95	−0.04	−0.04	−1.79, 1.72	−0.62 ***	−3.06	−1.02, −0.22
Age	−0.14 *	−1.70	−0.29, 0.02	0.13 **	2.05	0.00, 0.26	0.01 **	2.29	0.00, 0.03
Deprivation level	0.30	1.20	−0.19, 0.78	0.00	0.00	−0.39, 0.38	0.01	0.29	−0.03, 0.04
% green space (continuous)	−0.08 **	−2.28	−0.14, −0.01	-	-	-	−0.001	−0.21	−0.01, 0.01
binary green space (≥43%)	-	-	-	3.00 ***	2.79	0.86, 5.14	-	-	-
No Garden	3.48 **	2.06	0.13, 6.82	-	-	-	-	-	-
**Interaction Effects**									
No Garden with gender (female)	−4.31 *	−1.93	−8.75, 0.13	-	-	-	-	-	-
% green space (continuous) with gender (female)	-	-	-	-	-	-	0.01 **	2.05	0.00, 0.02

Note cells with “-” denotes terms removed from the model for that particular response variable. * *p* < 0.10; ** *p* < 0.05; *** *p* < 0.01.

Model 1

*Perceived Stress:* being female and living with lower neighbourhood green space was associated with higher perceived stress. Not having a garden was associated with higher stress levels but, as the interaction term shows, only for males (to clarify: the main effect of “no garden” for males corresponds to the main effect for “no garden”, and total effect for females is 3.48−4.31 = −0.83, t = −0.71, *p* = 0.683, hence not significant). The overall model was significant at *p* = 0.003 with adjusted r^2^ value of 13%.

Model 2

**Figure 1:**
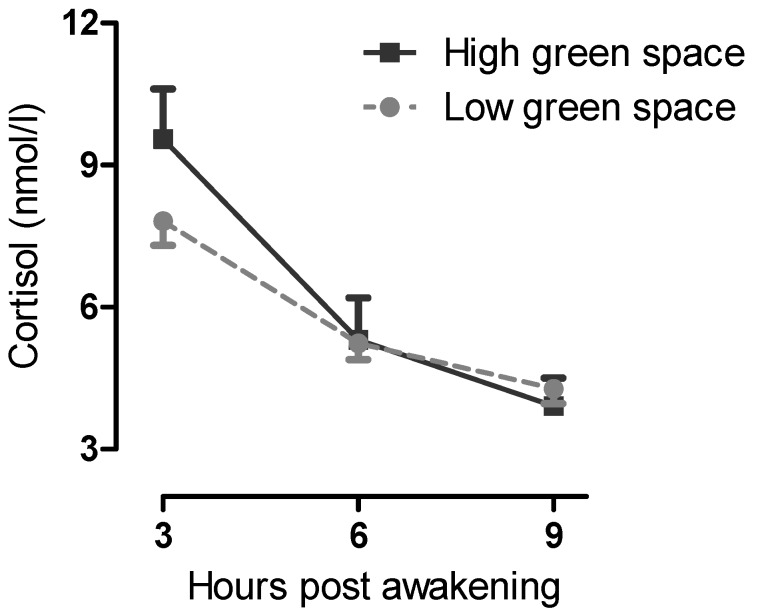
Difference in cortisol slope between participants living in high *versus* low green space areas.

*Cortisol Slope:* Increasing age and living with high rather than low levels of green space (binary variable) was associated with a greater diurnal cortisol decline. This pattern is illustrated in Figure 1 which shows that participants living in areas of higher green space had a steeper cortisol slope profile whilst those participants living with lower neighbourhood green space have a flatter slope profile.

There was no evidence of gender or interaction effects of gender with green space in the models, suggesting the positive association between green space and cortisol slope was consistent in both men and women. The overall model was marginally significant at *p* = 0.052 with adjusted r^2^ of 6.3%.

Model 3

**Figure 2:**
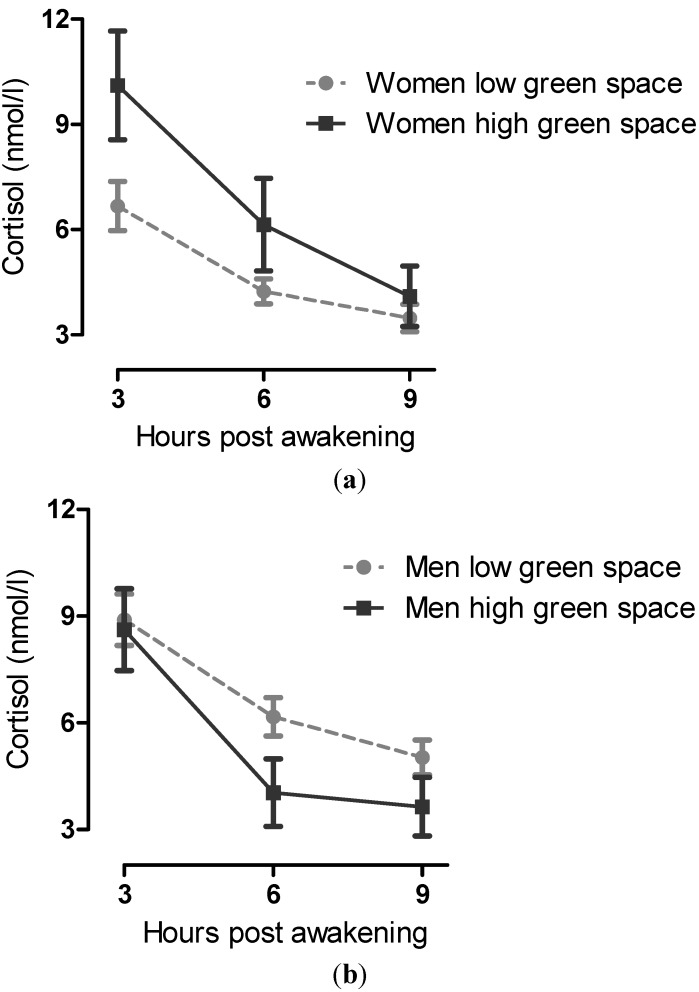
**(a)** Differences in mean cortisol slope in women living in high *versus* low green space areas. **(b)** Differences in mean cortisol slope in men living in high *versus* low green space areas.

*Cortisol Mean Concentration*: Being female and of younger age was associated with lower average cortisol concentrations (Table 4). There was no significant overall association between neighbourhood green space and average cortisol concentrations. There was a significant interaction between percentage green space and gender such that higher neighbourhood green space was associated with higher average cortisol levels among females, with no effect for males. The overall model was significant at *p* = 0.004 with adjusted r^2^ value of 13%. The interaction effect between gender and green space (F = 7.03, df = (1,94), *p* = 0.009) was confirmed in a repeated measures (multilevel) analysis (using the original separate cortisol readings within individuals), although we prefer to present the separate, simpler analyses here, as there are fewer problems caused by missing values. In low green space, women showed a “low and flat” slope, illustrated in Figure 2(a). For men, lower green space was associated with a “high flat” cortisol slope associated as illustrated in Figure 2(b).

In all three models, age was a significant predictor of stress: with increasing age perceived stress decreased and mean cortisol concentrations were higher with a healthier—and steeper—diurnal slope. We found no evidence of omitted variable bias in any of our three models.

### 3.2. Discussion

As hypothesised, the study found that higher levels of neighbourhood green space were associated both with lower levels of perceived stress (Hypothesis 1) and a steeper diurnal decline in cortisol secretion, for men and women (Hypothesis 2). Green space was found to be an independent predictor of the circadian cortisol cycle. This confirms the earlier study [22] and establishes further evidence of an association between levels of neighbourhood green space and neuroendocrine function, which may point to better understanding of any link between the observed beneficial effects of green space and health.

A new finding—and atypical of a more socially advantaged middle-aged population—was that lower cortisol levels were associated with flat diurnal cortisol profiles, suggestive of a prevalence of hypocortisolemia and chronic stress. Dysregulation of the diurnal pattern of cortisol secretion is associated with an array of negative health outcomes [31].

In addition, we found different levels of perceived stress and different cortisol secretion patterns in men and women (Hypothesis 3) that also varied by amount of green space in the local area. Firstly, women in low green space areas had significantly higher levels of perceived stress. Secondly, the diurnal cortisol pattern between men and women was different in areas of low green space but not in high green space areas. Women showed evidence of greater hypocortisolemia (*i.e.*, a “low flat slope” cycle) and men a “high flat slope” cycle in low green space areas. A low flat cycle is associated with exhaustion and chronic stress [19,32,33,34], whereas a high flat cycle—whilst also indicative of severe stress—suggests better overall stress regulation [35,36]. This finding is consistent with reports of hypocortisolemia in middle-aged females exposed to stress, depression and negative life events [17,18,19]. It suggests that high green space may contribute to lessening the differential effect of stress on women compared with men. Whilst associations between stress levels and access to gardens were not the focus of this study, it is interesting to note that perceived stress was higher for those with no garden, but that this effect was significantly weaker for females. This suggests further research should explore types of green space, including access to gardens, and gender effects on stress levels.

We found a positive relationship between higher green space levels and mean cortisol levels in women, but not in men. It seems that higher neighbourhood green space may contribute in some way to higher but healthier cortisol levels in this female population and, conversely, to moderating unhealthily high cortisol levels in men (*i.e.*, cortisol concentrations in men come down in higher green space areas to a healthier level). This finding provides further evidence of gender differences in neuroendocrine associations with psychosocial factors and points to gender differences in the neuroendocrine response to the environment. However it is interesting that in males and females, irrespective of overall cortisol levels, a greater diurnal decline in cortisol was associated with more neighbourhood green space. High green space levels therefore appear to offer some degree of stress buffering in this socially disadvantaged population, reducing or moderating the differences in stress levels perceived in men and women compared with those living with low green space.

The participants in this study were mostly middle-aged and long-term residents in urban, deprived neighbourhoods. It is plausible that the impact of unemployment and long-term socio-economic adversity in the participants resulted in long-term HPA axis dysregulation and the cortisol profiles presented here. Recently it has been shown that adult only city living is associated with greater stress-related activity in the amygdala, which activates the HPA axis [37], Unfortunately, the impact of lifetime exposure to city dwelling was not directly explored in this study although, in the neighbourhoods surveyed, it is typical for residents to have been life-long inhabitants of the same city. For the purpose of this current study, what is of most interest is that those residing in city areas with higher levels of green space report less perceived stress and appear to have been more resilient to the negative effects of urban deprivation as evidenced by a steeper cortisol slope profile.

These results are potentially important in understanding how neighbourhood green space might contribute to public health improvement. Stress is known to impact on cardiovascular disease via interaction with a variety of risk factors that include genetics, early experience, age, race, diet and physical activity [38], with women at greater risk [39,40] but little is known about the environmental factors that might also contribute. At a population level, higher levels of green space are associated with reduced cardio-vascular mortality in England and Wales [12]. Our data indicate that neighbourhood green space is associated with perceptions of stress as well as the stress neuroendocrine system and this may be a pathway by which the environment can impact health. Whilst more research is needed to understand these mechanisms, including a better understanding of how use of nearby green space may affect the pattern of stress associated with higher neighbourhood green space, our study represents a valuable step in establishing a biological pathway linking green space with stress levels in deprived urban environments.

### 3.3. Limitations

Our study was cross-sectional in design and therefore cannot demonstrate causality. Whilst demographic and socio-economic factors were accounted for, it is possible that other individual and environmental factors, for example smoking status, or social contact, may have influenced outcomes. The percentage of smokers in our sample was—based on pilot screening—known to be high, with smoking levels in deprived areas of Dundee over 50% [41]. It was not realistic, therefore, in this population, to exclude smokers from our study. Whilst smoking increases levels of salivary cortisol it has a short-term effect on the neuroendocrine system. We controlled for this with text prompts—issued to participants at timed intervals during the day advising them to refrain from smoking (and other stimulants) half an hour prior to sampling procedures. We have no reason to believe participants did not comply with this instruction: the linear decline data suggests they did not smoke—as requested—otherwise we would have seen a spike in cortisol. All spikes detected were managed in the data set as described in Section 2.6.

We were unable to confirm the significant relationship found in the earlier study between cortisol slope and physical activity, although we found a positive correlation between these two variables, showing a positive effect of increased physical activity on cortisol slope patterns. We suspect this is owing to a high proportion of missing data in answering the physical activity question (12% of our sample did not provide data for this variable). In future, more objective measures might usefully be used to establish daily physical activity levels in participants e.g., accelerometers. We would also recommend the inclusion of a social connectedness scale to further explore relationships between stress and levels of green space but—in the current study—this was outwith our agenda.

We chose to examine the diurnal decline in cortisol secretion (3, 6 and 9 h post awakening) as opposed to the cortisol awakening response (CAR). It was not practical in this deprived population to impose the intensive post awakening saliva sampling regime required for determination of the CAR. In addition we did not have objective means of verifying adherence with the required regime which is essential for accurate determination of this measure (recently it has been shown that post awakening non-adherence of just 5–15 min can lead to misleading CAR estimates) [42]. Diurnal cortisol patterns have been established using as few as two post-awakening samples (at 3 and 6 h); establishing the slope pattern over the day between 3–9 h post awakening was therefore felt to be an appropriately rigorous measurement in this context.

Owing to limitations in UK land use classifications systems our objective measure of percentage green space was unable to pick up the finer grain detail of front gardens and street trees. Improving the measurement of different types of green space is therefore one important area to develop in future research to test hypotheses in relation to gender differences in relation to associations between access to private/semi-private gardens, other types of green space and health.

## 4. Conclusions

Higher levels of neighbourhood green space in deprived urban communities are linked with lower perceived stress and a steeper diurnal decline in cortisol secretion, confirming the earlier study [22]. This new study extends the evidence for a biological pathway which may explain the link between neighbourhood green space and health outcomes, for which stress has an aetiological role. In addition, we found evidence of hypocortisolemia (*i.e.*, lower daytime levels of cortisol) in this socially deprived urban population, an atypical pattern and indicative of chronic stress, with greater hypocortisolemia evident in women living in low green space. Furthermore, we have shown that higher levels of green space are associated with a lower level of this pattern in women, with a positive association of increased green space with mean cortisol concentrations, warranting further investigation. Through successful replication of earlier methodology, we have demonstrated that this experimental approach is an ecologically valid and reliable method for furthering evidence of salutogenic environment-body interactions within a real-world context.
